# Satisfaction and continuation intention in music streaming services: investigating key factors for user retention

**DOI:** 10.3389/fpsyg.2025.1552800

**Published:** 2025-06-04

**Authors:** Qianwen Li, Yapu Liu, Chen Chen

**Affiliations:** ^1^College of Music and Dance, Xihua University, Chengdu, Sichuan, China; ^2^School of Music and Dance, Aba Normal College, Chengdu, Sichuan, China

**Keywords:** music streaming, continuance intention, service satisfaction, user retention, expectation confirmation

## Abstract

**Background:**

The business landscape for music streaming services has shifted significantly over the past decade. Retaining music streaming users is critical for service providers' sustainability.

**Methods:**

This study utilized an electronic questionnaire to survey 895 music streaming users. Structural equation modeling was conducted using Smart-PLS software to investigate the factors influencing service satisfaction and continuation intention among music streaming users. This study is grounded in the Expectation Confirmation Model and builds upon the theoretical foundation of the Information System Success Model as well as prior research. To enhance the analytical framework, four key variables have been incorporated: information quality, system quality, service quality, and perceived fair pricing. This extension aims to provide a more comprehensive understanding of the factors influencing user satisfaction and continuation intention, thereby offering a systematic theoretical framework for examining user behavior within the context of music streaming services.

**Results:**

The study showed that service satisfaction plays a central role in driving continuation intention, emphasizing its importance for user retention. Perceived usefulness was found to significantly impact service satisfaction, which in turn influenced users' intention to continue using the service. Expectation confirmation was a key determinant of service satisfaction, but it did not directly affect perceived usefulness, suggesting that user expectations in the context of music streaming services are more influenced by content availability and user experience than by platform functionality. Additionally, perceived fair pricing, information quality, and system quality all contributed to higher levels of service satisfaction and perceived usefulness, reinforcing their critical role in enhancing user engagement and retention in a competitive market.

**Conclusion:**

This study can provide guidance for music streaming service providers to improve user retention and payment rates.

## 1 Introduction

With the rapid advancement of Internet technology and the global popularity of smart mobile devices, music streaming services have emerged and quickly transformed from an emerging music consumption model to a mainstream trend in the global music industry (Arthur et al., 2023). By providing real-time, convenient access to a vast library of music, music streaming not only satisfies the modern consumer's desire for instantaneous and personalized music experiences but also completely reshapes the landscape of the music industry (Barata and Coelho, 2021). The beauty of music streaming lies in its unlimited accessibility and personalized recommendations, allowing users to explore and enjoy the rich diversity of music anytime, anywhere. As service providers continue to enhance the user experience through innovative means such as data analytics, user customization, and social interaction, music streaming not only shows great business potential but is also expected to become the core force driving the development of the global music market and leading the new trend of music consumption (Ranasingha and Silva, 2023).

Despite the widespread support for music streaming services' business prospects, it is crucial to acknowledge the intense competition in the market. Numerous service providers are constantly innovating their services in order to compete for market share, trying to attract and retain users by offering unique user experiences (Hracs and Webster, 2021). Spotify, for example, converts users through a “freemium” model, while Apple Music relies on exclusive content to target high-end subscribers (Carroni and Paolini, 2020). However, the business model of music streaming is still in the exploratory stage, and service providers are even more faced with the key challenge of user lifecycle management—from first contact to paid conversion to long-term retention, each stage involves complex psychological decision-making mechanisms (Clauss et al., 2019).

Service satisfaction (SS) and continuation intention (CI) are critical indicators of music streaming service providers' profitability and market resilience (Hu et al., 2020). Both are not only barometers of the financial health of a platform but also visual reflections of the service quality (SQ) and market attractiveness of the platform (Dai et al., 2022). The growing popularity of music streaming services has prompted extensive research into the factors influencing user satisfaction and retention (Lin and Bowman, 2022). Previous studies have predominantly applied established models, such as the Technology Acceptance Model and the Expectation Confirmation Model, to investigate user adoption and continued usage behaviors (Li et al., 2024). While these models have provided valuable insights, they often conceptualize satisfaction as a unidimensional construct, overlooking its potential multidimensionality. According to Burkov et al. (2023), satisfaction can encompass affective, cognitive, and hedonic dimensions, which together contribute to a more comprehensive and nuanced understanding of user experiences than a unidimensional approach allows. Moreover, despite the frequent adoption of variables such as perceived usefulness (PU) within both the Technology Acceptance Model and the Expectation Confirmation Model, there is a persistent issue of conceptual ambiguity and inconsistency. While PU is a core construct in both models, its interpretation differs: in the Technology Acceptance Model, it primarily refers to the degree to which using a system enhances task performance (Li et al., 2024), whereas in the Expectation Confirmation Model, it is often framed in terms of the fulfillment of user expectations and overall satisfaction (Dhiman et al., 2022). However, many studies fail to distinguish these nuances, instead employing PU as a generic construct without recognizing its differing theoretical underpinnings across models (Kumar and Natarajan, 2020). Furthermore, existing research has often confined its scope to a limited set of variables, primarily emphasizing content quality or isolated technical features, while neglecting the broader construct of perceived fair pricing (PFP; Schiebler et al., 2025).

In this context, the Expectation Confirmation Model provides a valuable theoretical foundation, encompassing key variables such as PU, SS, expectation confirmation (EC), and CI. However, to gain a more comprehensive understanding of user retention, it is essential to include additional factors such as information quality (IQ), system quality (SysQ), SQ, and PFP (DeLone and McLean, 2003; Schiebler et al., 2025). These variables offer a more nuanced perspective on how both content-related and technical aspects contribute to users' overall experiences, shaping their satisfaction and their intention to continue using the service. By broadening the theoretical framework to incorporate these additional dimensions, this research enhances our understanding of the multifaceted factors driving user satisfaction and retention in music streaming services. This expanded viewpoint offers critical insights for service providers aiming to improve customer loyalty and optimize their offerings for sustained success.

## 2 Literature review and research hypotheses

To understand the factors influencing user retention in music streaming services, this study is grounded in the Expectation Confirmation Model as the core theoretical framework. At the same time, to address the limitations of the Expectation Confirmation Model, we incorporate the Information System Success Model and the insights from prior research. The details of these theories are presented as follows.

### 2.1 Expectation confirmation model

Bhattacherjee (2001) proposed the Expectation Confirmation Model, a theoretical framework that examines how users develop attitudes and behaviors conducive to the sustained use of information systems following their initial adoption. The Expectation Confirmation Model is grounded in Expectancy Confirmation Theory, which has been widely used to explore consumer satisfaction and behavioral continuity. According to Expectancy Confirmation Theory, consumer repurchase decisions are influenced by whether the product or service confirms their initial expectations, which in turn leads to satisfaction. This model, therefore, emphasizes that when a system's actual performance meets or exceeds a user's expectations, the user experiences a sense of confirmation, which fosters positive emotions.

The Expectation Confirmation Model has been widely applied to explore the relationship between consumer satisfaction and behavioral continuance intentions (Rahi and Abd. Ghani, 2019; Tam et al., 2020). This model consists of four core variables: PU, confirmation, satisfaction, and continuance intention (Wang and Wang, 2019). Within this framework, confirmation represents the cognitive process through which consumers compare their initial expectations with actual experiences, and it directly influences both PU and satisfaction. Specifically, PU impacts consumers' evaluations of the product or service's effectiveness, which in turn affects their overall satisfaction and their intention to continue using the service. Furthermore, satisfaction plays a critical role in the decision-making process, as it not only reflects consumers' overall evaluation of the product or service but also directly influences their future intentions to continue usage. Therefore, the Expectation Confirmation Model provides a valuable perspective by elucidating the complex dynamics between consumer cognitive evaluations and subsequent behavioral choices through the interplay of these four core variables.

Adopting the Expectation Confirmation Model as the central framework for this study is both appropriate and effective. From a theoretical perspective, the model provides a robust foundation for understanding how users' expectations influence their satisfaction and continuance intentions. Empirically, prior research has demonstrated the model's applicability in predicting consumer behavior in various service contexts, making it an ideal fit for our investigation. In this research, the Expectation Confirmation Model is applied to examine how music streaming service users' initial expectations, formed based on service offerings, are confirmed or disconfirmed through actual use. The confirmation process shapes their PU of the service and, consequently, their overall satisfaction. This satisfaction, in turn, influences their intention to continue using the service. This model enables a nuanced understanding of user retention, providing insight into how service providers can better align user expectations with actual service experiences to enhance satisfaction and encourage continued engagement.

In addition, consistent with the work of Burkov et al. (2023), satisfaction is conceptualized as comprising three distinct dimensions: emotional, cognitive, and hedonic. This multidimensional approach allows for a more comprehensive and nuanced understanding of user experience, recognizing that satisfaction is not a unidimensional construct but rather a complex interplay of affective, cognitive, and pleasurable elements. Each of these dimensions plays a pivotal role in shaping the overall user experience, offering valuable insights into how individuals engage with a service on multiple levels. Given this conceptualization, the variable of SS in this study is measured across these three dimensions. The following are a few hypotheses based on Expectation confirmation model:

H1. Users' EC of the music streaming positively influences PU. Once users' expectations are confirmed, they are more likely to perceive the service as providing convenience, entertainment, or other forms of value (EC → PU).
H2. Users' EC of the music streaming positively affects SS, which means that if users' experience of the service meets or exceeds their original expectations, their SS will also increase (EC → SS).
H3. Users' PU toward music streaming positively enhances SS. Their overall satisfaction with the service will be stronger if it is perceived as effective in meeting users' needs (PU → SS).
H4. Users' SS toward music streaming positively influences CI. Satisfied users are more likely to maintain usage, demonstrating loyalty to the service (SS → CI).

### 2.2 Expansion variables

#### 2.2.1 IQ, SysQ and SQ

The Information System Success Model is a widely used framework for defining and assessing the success of information systems (DeLone and McLean, 2003). It offers a comprehensive approach to evaluating how well information systems meet users' needs and expectations, and it has become a foundational model in the field of information systems research (Chen and Tsai, 2019). According to the ISSM, the success of an information system can be explained through three key dimensions: IQ, SysQ, and SQ (DeLone and McLean, 2003).

IQ refers to the extent to which the information provided by the system is accurate, timely, relevant, and useful for the users (Laumer et al., 2017). In the context of this study, IQ specifically pertains to the music content offered on a music streaming platform, emphasizing factors such as the variety, accuracy, and relevance of the music library available to users.

SysQ pertains to the technical performance and functionality of the system. This includes aspects such as system reliability, ease of use, responsiveness, and overall performance (Gupta and Bhatt, 2021). In this study, SysQ specifically refers to the operational framework and technological infrastructure underlying the music streaming platform, including factors like the stability of the platform, user interface design, and the efficiency of content delivery.

SQ refers to the overall quality of the support and services provided to users, including customer service, technical support, and additional services that enhance the user experience (Gupta, 2016). In this research, SQ specifically refers to the various services offered by the music streaming platform to its users, including features such as customer support, account management, content personalization, and other user-centric services that contribute to overall user satisfaction.

The quality of an information system plays a pivotal role in shaping users' perceptions of its usefulness (Petter et al., 2008). Consequently, users' PU can be conceptualized by evaluating various dimensions of SysQ (Salim et al., 2021). In this study, we adopt IQ, SysQ, and SQ as second-order constructs to represent consumers' PU while using the music streaming service. This approach directly incorporates the quality factors embedded within the Expectation Confirmation Model, offering a nuanced understanding of how these quality dimensions collectively influence users' perceptions of the service's value. By focusing on these specific dimensions, we can better capture the complex interactions that determine user satisfaction and contribute to continued engagement with the platform. This methodology allows for a comprehensive assessment of the factors driving users' perceptions of the streaming service's effectiveness, ultimately offering insights into the elements that enhance user retention and loyalty. This study proposes the following hypothesis based on the information system success model:

H5. Users perceive a music streaming service as more useful if the information provided by the platform is of high quality, characterized by accuracy, relevance, and timeliness (IQ → PU).
H6. Users perceive a music streaming service as more useful if the system underlying the platform is reliable, user-friendly, and efficient in terms of performance and functionality (SysQ → PU).
H7. Users perceive a music streaming service as more useful if the platform provides high-quality customer service, including responsive support and personalized user assistance (SQ → PU).

Furthermore, when users' expectations are based on the quality of information they expect to receive from a service, IQ serves as a key determinant in whether these expectations are confirmed or disconfirmed. According to expectancy-confirmation theory, users form initial expectations about a service based on various sources, such as marketing materials, user reviews, and prior experiences. The perceived quality of the information provided by the service plays a pivotal role in whether users' actual experiences align with or deviate from these expectations. When the music streaming service provides high-quality information—such as accurate song metadata, well-curated playlists, and timely updates to the catalog—users are more likely to experience positive EC. This occurs because the information provided by the service matches or exceeds the users' initial expectations. For example, if a user expects a wide range of high-quality music and the streaming service delivers an extensive and up-to-date library, the user's expectation is confirmed, reinforcing their belief that the service meets their needs. This alignment between expectations and actual service performance enhances the overall user experience, fostering satisfaction and continued engagement with the platform. Therefore, based on the preceding discussion, we propose the following hypothesis:

H8. High-quality information provided by the music streaming service leads to a greater alignment between users' initial expectations and their actual experiences, thereby confirming their expectations (IQ → EC).

#### 2.2.2 PFP

PFP refers to consumers' subjective assessment of the reasonableness of the price of a product or service, which is determined through a comparison between the price paid, the value received, and the prices paid by other consumers (Shaw et al., 2022). In the context of music streaming services, PFP includes users' perceptions of subscription fees, song purchase prices, and other associated costs. These perceptions have significant implications for both EC and satisfaction.

When consumers perceive the price of a music streaming service as fair, it aligns with their initial expectations regarding the value they expect to receive in exchange for the price paid. Positive disconfirmation occurs when the service's performance meets or exceeds these expectations, confirming that the value derived from the service justifies the price (Hufnagel et al., 2022). For instance, if a user expects to receive access to a vast library of music and high-quality audio at a reasonable price, and the service fulfills these expectations, it can lead to positive confirmation, reinforcing the perceived fairness of the pricing structure (Jia et al., 2023). Conversely, if consumers perceive the price as unfair—either because the service does not deliver the expected value or because they feel they are paying more than what others are paying for similar services—expectation disconfirmation becomes negative (Kalyanaram et al., 2022). This negative disconfirmation arises when the actual value received is less than anticipated, leading to dissatisfaction with the pricing, which undermines the user's confidence in the fairness of the service (Bambauer-Sachse and Young, 2024). The perceived unfairness of the pricing structure, in turn, can negatively affect the users' perceptions of the service's overall value and their continued use intentions.

PFP plays a critical role in shaping overall satisfaction with music streaming services. When consumers perceive the pricing to be fair, they are more likely to express positive satisfaction with the service, as the perceived value aligns with their expectations (Zietsman et al., 2019). The fairness of the price enhances the users' overall satisfaction because they feel they are getting a reasonable return on their investment, fostering a positive attitude toward the service. Studies have shown that consumers who perceive fair pricing are more likely to exhibit higher satisfaction levels, which often leads to increased loyalty and greater likelihood of recommending the service to others (Jones et al., 2019). On the other hand, when the price is perceived as unfair, users are likely to feel that the service does not provide sufficient value for the price they are paying (Riquelme et al., 2019). This dissatisfaction can reduce their overall satisfaction and lower their willingness to continue using the service. Perceived unfair pricing can also lead to negative behavioral intentions, such as the intention to switch to competing services that offer better value, further diminishing the user's satisfaction with the current service (Eyster et al., 2021). In essence, PFP directly impacts satisfaction by influencing the perceived value of the service relative to its cost, and this relationship plays a central role in determining user loyalty and retention.

Incorporating PFP into the Expectation Confirmation Model and other theoretical frameworks enhances their comprehensiveness by acknowledging the importance of cost-benefit considerations. Unlike the Technology Acceptance Model and the Expectation Confirmation Model, which typically overlook pricing factors, the inclusion of PFP allows for a more nuanced understanding of how pricing strategies influence user satisfaction and continuance intentions (Wang and Wang, 2019). Thus, it could be hypothesized that:

H9: PFP influences EC, such that when users perceive the price of a music streaming service as fair, it aligns with their initial expectations and leads to positive EC (PFP → EC).
H10: PFP influences satisfaction, such that when users perceive the price as fair, they are more likely to experience higher satisfaction with the music streaming service. Conversely, when the price is perceived as unfair, it leads to lower satisfaction (PFP → SS).

## 3 Materials and methods

### 3.1 Research design

**Figure 1:**
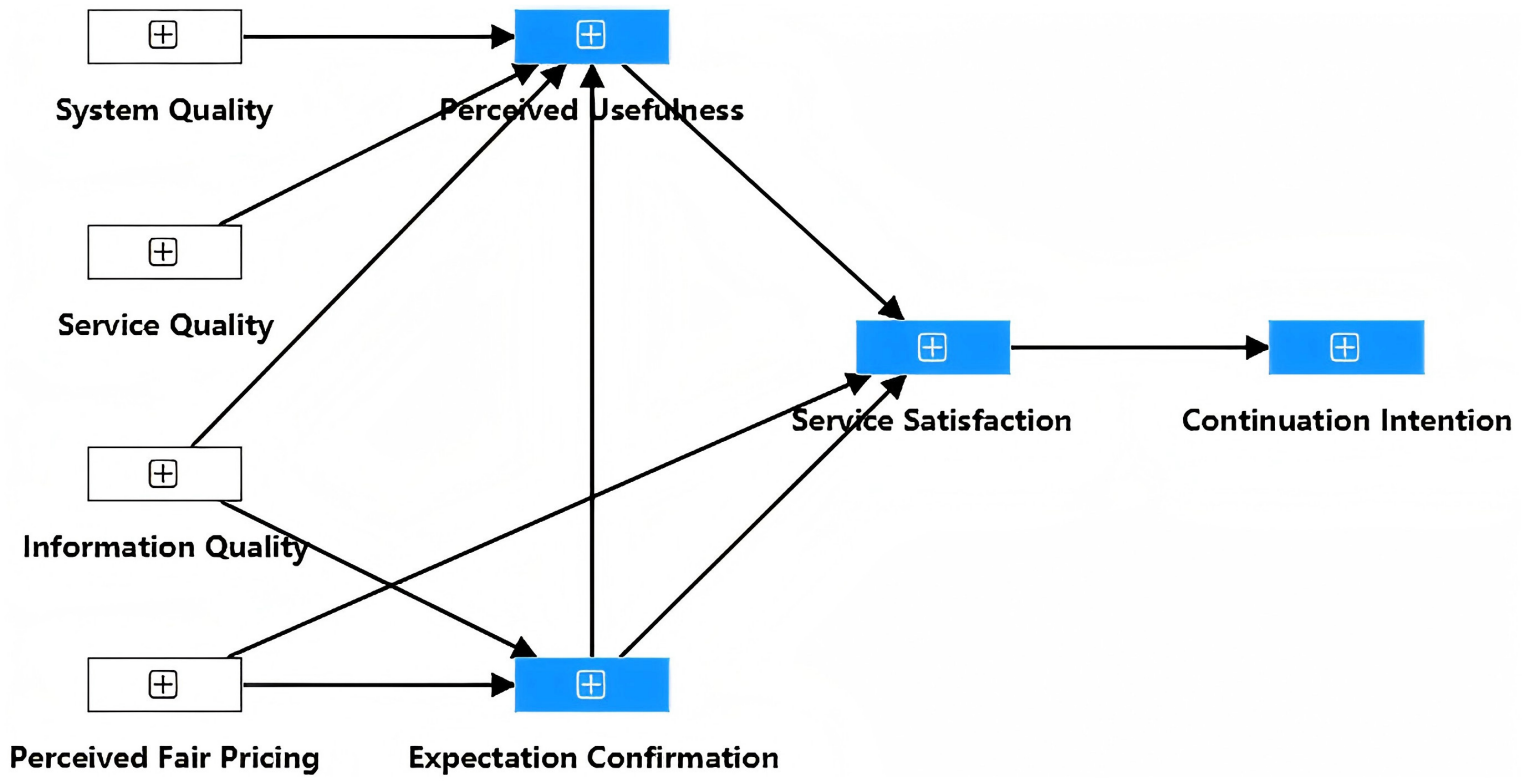
Hypothetical model.

This study adopts the expectation confirmation model as the core theoretical framework, extending it with four additional variables to create a SEM comprising eight variables. The model, as illustrated in Figure 1, seeks to examine the factors influencing user satisfaction and CI within the context of music streaming services.

To measure the variables within the proposed model, this study developed measurement instruments through the adaptation of previously validated scales, ensuring their relevance to the current research context. Specifically, the measurement instruments were modified to better reflect the unique features of music streaming services. The structure of the measurement instruments consists of multiple-item scales, each of which was adapted from established literature and further revised to align with the context of this study. The items measuring PU, SS, EC, and CI were adapted from Bhattacherjee (2001), who provided validated scales in the context of information systems. These constructs are essential in understanding the cognitive and emotional responses of users as they interact with music streaming platforms. The items related to IQ, SysQ, and SQ were adapted from the work of DeLone and McLean (2003), whose research on information systems success provided a comprehensive foundation for measuring the quality dimensions that are critical to user experience. Lastly, the items measuring PFP were adapted from Shaw et al. (2022), addressing the relevance of pricing perceptions in consumer decision-making.

The measurement instruments utilized in this study incorporated demographic questions, along with multiple items designed to assess each variable in the model. To ensure the reliability and validity of respondents' attitudes and perceptions, an 11-point semantic differential scale was employed to rate each item. The scale ranged from 1 (extremely negative) to 11 (extremely positive), offering a broader spectrum for capturing the intensity of respondents' views and emotions regarding each construct. This scale is particularly advantageous in survey-based research as it allows for a more refined measurement of subjective opinions and attitudes, facilitating a more nuanced evaluation of participants' responses. The use of the 11-point semantic differential scale enhances the precision of the data analysis, enabling a more robust interpretation of the results.

### 3.2 Participants and data collection

This study utilized a stratified random sampling approach through the Wenjuanxing platform, selecting samples from the active user pool within the database based on demographic factors (age, gender, and region), with 30% of the sample specifically allocated to inactive users of music streaming platforms. To ensure the representativeness of the sample, participant account types (free/premium) and user statuses (new users/existing users) were distinctly identified, and geographic distribution was verified through IP addresses.

A dual screening mechanism was adopted: first, the system automatically removed 12 incomplete questionnaires; subsequently, the research team manually reviewed 45 rapidly completed responses (completion time <60 s). The review criteria were based on the response quality assessment framework proposed by Dudek (2019), integrating attention-check questions and logical consistency validations. Furthermore, the average questionnaire completion time (M = 4.2 min, SD = 1.3) was recorded as an auxiliary indicator of data quality. This completion duration showed significant consistency with preliminary experimental results (M = 4.1 min, SD = 1.1) (*p* = 0.82).

The final dataset comprised 895 valid questionnaires, structured into two distinct modules. The first module collected general demographic information, including age (M = 28.5, SD = 8.3), gender (male 47.6%, female 52.4%), and higher education attainment (82.1%). The second module assessed 11 variables related to music streaming platforms, using a 5-point Likert scale to capture respondents' attitudes and perceptions. During the data processing phase, differential privacy techniques (Arif et al., 2021) were applied to anonymize sensitive information such as occupation through k-anonymity (*k* = 5). Additionally, data masking techniques were employed to ensure the confidentiality and untrace ability of user behavior trajectories.

### 3.3 Data analysis

This study provides an in-depth analysis of the key factors influencing users' SS and CI to music streaming through a combination of partial least squares structural equation modeling (PLS-SEM), bootstrapping, and blindfolding techniques. Using the power of SmartPLS software, the study aims to deconstruct and understand the complexity of user behaviors in the music streaming domain. The combination of the three approaches provides a comprehensive analytical framework for this study to unpack the key drivers influencing users' SS and CI to music streaming services with a high degree of confidence and validity.

Firstly, the application of PLS-SEM allows for a fine-grained exploration and validation of causal models, and its ability to deal with complex models is particularly outstanding. Even when the data sample size is small or the distribution does not fully conform to the normal distribution, it is also capable of outputting reliable analysis results (Chin et al., 2020). Through the initial run of PLS-SEM, the study reveals the degree of interaction and influence between multiple variables.

Then, the statistical significance of the variables was rigorously tested through the bootstrapping procedure to ensure the trustworthiness of the analyses (Razak et al., 2018). Finally, the assessment of the predictive power of the model was enhanced by the blindfolding procedure, which ensured that the constructed model was able to effectively predict user behaviors, a process that provides valuable information for optimizing music streaming services (Shmueli et al., 2019).

## 4 Results

### 4.1 Measurement model

#### 4.1.1 Reliability

When conducting reliability tests for structural equation modeling, it is usually necessary to assess both indicator reliability and construct reliability. Indicator reliability is the extent to which individual observations reflect the constructs they represent. Construct reliability, on the other hand, involves an evaluation of the internal consistency of the construct.

Indicator reliability was assessed through the outer loadings of the observed variables. The outer loadings reflect the strength of the correlation between each observed indicator and the latent variable it represents. According to Götz et al. (2009), an indicator is considered to have a strong correlation with its latent variable if its loadings are greater than 0.7. The results of this study showed that the loadings of all the indicators ranged from 0.718 to 0.912, which exceeded the recommended threshold of 0.7, thus indicating that each indicator was significantly correlated with its corresponding latent variable.

Cronbach's alpha is the most widely used indicator of internal consistency and reliability, with higher values indicating better inter-item agreement within the scale (Kalkbrenner, 2023). A Cronbach's alpha value of 0.7 or above is generally considered to be an acceptable level of reliability. In this study, the Cronbach's alpha values for all variables were above 0.847, which indicates that the construct scales performed well in terms of internal consistency (see Table 1).

**Table 1:** Construct reliability and validity.

**Construct**	**Cronbach's alpha**	**Composite reliability (rho_a)**	**Composite reliability (rho_c)**	**Average variance extracted (AVE)**
CI	0.915	0.917	0.937	0.747
CQ	0.875	0.878	0.910	0.669
EC	0.922	0.923	0.942	0.764
PFP	0.967	0.967	0.971	0.791
PU	0.956	0.957	0.962	0.677
SQ	0.847	0.852	0.897	0.686
SS	0.958	0.959	0.962	0.614
TQ	0.924	0.926	0.943	0.767

In addition, the composite reliability presented validates construct reliability through the composite reliability (rho_a) and composite reliability (rho_c) values. These values take into account the effect of measurement error and provide a more rigorous assessment of reliability. Composite reliability values above 0.7 are considered to indicate good reliability. The composite reliability (rho_a ≥ 0.852) and composite reliability (rho_c ≥ 0.897) values reported in this study exceeded this threshold, which reinforces the strength and stability of the scale in terms of construct reliability.

#### 4.1.2 Validity

Convergent and discriminant validity are two key aspects of evaluating the validity of latent variable constructs. Convergent validity focuses on whether the indicators for a single construct reflect that construct consistently enough, while discriminant validity focuses on ensuring that different constructs are clearly distinguishable from one another.

The test of convergent validity can be implemented through Average Variance Extracted (AVE). The AVE value measures the variance extracted by construct indicators and assesses how well the indicators collectively reflects the construct. An AVE value above 0.5 is considered acceptable, implying that a large portion of the variance of the construct is explained by its indicators. The AVE values (AVE ≥ 0.614) for all latent variables in this study exceed this threshold, indicating that each latent variable exhibits good convergent validity. This result supports the hypothesis that the indicators are representative of the constructs.

The assessment of discriminant validity can be done through the Fornell-Larcker criterion and the heterotrait-monotrait ratio (HTMT).

The Fornell-Larcker criterion works by comparing the square root of the AVE of a latent variable to the maximum value of the correlation between that latent variable and other latent variables. If the AVE square root of a construct is greater than the correlation coefficient with any of the other constructs, this indicates sufficient differentiation between the different constructs (De Silva, 2023). In Table 2, the AVE square root (values on the diagonal) for each latent variable is greater than the correlation coefficient between the corresponding latent variable and the other latent variables, thus satisfying the Fornell-Larcker criterion and demonstrating good discriminant validity.

**Table 2:** Fornell-Larcker.

	**CI**	**CQ**	**EC**	**PFP**	**PU**	**SQ**	**SS**	**TQ**
CI	0.864							
CQ	0.507	0.818						
EC	0.670	0.455	0.874					
PFP	0.724	0.377	0.675	0.889				
PU	0.481	0.709	0.381	0.325	0.823			
SQ	0.421	0.517	0.381	0.293	0.680	0.828		
SS	0.781	0.523	0.727	0.738	0.493	0.422	0.783	
TQ	0.493	0.760	0.432	0.294	0.721	0.567	0.484	0.876

HTMT is a relatively recent approach used to assess discriminant validity, which compares the correlations between different constructs (heterogeneous features) to the correlations within the same construct (homogeneous features). HTMT values below 0.85 typically indicate strong discriminant validity, although some studies accept values up to 0.90. In Table 3, the HTMT values between all latent variables were below the threshold of 0.845, indicating good discriminant validity between the constructs. It is important to note that these values consistently remained well within the acceptable range, further supporting the robust discriminant validity of the model.

**Table 3:** Discriminant validity-HTMT.

	**CI**	**CQ**	**EC**	**PFP**	**PU**	**SQ**	**SS**	**TQ**
CI								
CQ	0.568							
EC	0.729	0.508						
PFP	0.769	0.413	0.714					
PU	0.515	0.773	0.406	0.340				
SQ	0.482	0.598	0.435	0.330	0.752			
SS	0.846	0.584	0.769	0.759	0.527	0.476		
TQ	0.541	0.843	0.472	0.315	0.765	0.637	0.531	

To ensure the robustness of the measurement model and reduce the risk of multicollinearity, variance inflation factor (VIF) values were calculated for all constructs. As shown in Table 4, the VIF values for the latent variables ranged from 1.000 (SS) to 2.648 (TQ), all well below the commonly accepted threshold of 3.0 (Shmueli et al., 2019). This indicates that the constructs are sufficiently distinct and free from significant multicollinearity issues, further supporting the reliability and validity of the measurement model.

**Table 4:** Collinearity statistics (VIF).

	**CI**	**CQ**	**EC**	**PFP**	**PU**	**SQ**	**SS**	**TQ**
CI								
CQ			1.166		2.524			
EC					1.321		1.939	
PFP			1.166				1.855	
PU							1.181	
SQ					1.549			
SS	1.000							
TQ					2.648			

### 4.2 Structural model

#### 4.2.1 R^2^

The R-square is an important statistic used to assess the extent to which the independent variables of a model explain the changes in the dependent variable. The R-square value of CI is 0.626, SS is 0.681, EC is 0.502, and PU is 0.667. It indicates that the model has a certain degree of explanatory power.

Meanwhile, the adjusted R-squared values of EC, PU, SS and CI are highly consistent with the original R-squared values, indicating that the increase in the number of independent variables does not lead to a spurious expansion of the explanatory power, reflecting the robustness of the research design and the appropriateness of the variable selection. This suggests that the study has appropriately controlled the complexity of the model to avoid oversimplification that ignores important variables and complexity that leads to overfitting.

#### 4.2.2 Q^2^

The Q^2^ value assesses how well the model can predict endogenous constructs. The endogenous constructs in this study include CI (Q^2^ = 0. 464), SS (Q^2^ = 0. 412), EC (Q^2^ = 0. 380), PU (Q^2^ = 0. 448), and SS (Q^2^ = 0.368). Each value is greater than 0, indicating that the model has reasonable validity for explaining and predicting key endogenous constructs.

#### 4.2.3 Path coefficients

This study employed Partial Least Squares (PLS) with bootstrapping procedures to assess the significance of the path coefficients and the explanatory power of the structural model. This methodological approach allows for robust estimation of relationships among variables while accounting for potential sampling variability, thus providing reliable insights into the model's validity and predictive capacity. Path coefficients reflect the strength of the direct effect of the independent variable (predictor variable) on the dependent variable (outcome variable). In structural equation modeling, these coefficients represent the causal relationship between one latent variable and another. Path coefficient results in Table 5 indicate that the data supports 9 out of the 10 research hypotheses.

**Table 5:** Path coefficients.

**Hypotheses**	**Path**	**Original sample**	**Sample mean**	**Standard deviation**	**T statistics**	***P* values**	**Results**
H1	EC -> PU	−0.025	−0.024	0.028	0.890	0.374	N/A
H2	EC -> SS	0.356	0.357	0.044	8.068	0.000	Supported
H3	IQ -> EC	0.234	0.234	0.033	7.092	0.000	Supported
H4	IQ -> PU	0.314	0.314	0.042	7.412	0.000	Supported
H5	PFP -> EC	0.587	0.588	0.031	18.767	0.000	Supported
H6	PFP -> SS	0.427	0.426	0.037	11.569	0.000	Supported
H7	PU -> SS	0.219	0.218	0.026	8.515	0.000	Supported
H8	SQ -> PU	0.286	0.287	0.044	6.523	0.000	Supported
H9	SS -> CI	0.791	0.793	0.019	41.065	0.000	Supported
H10	SysQ -> PU	0.365	0.365	0.035	10.560	0.000	Supported

For the core framework Expectation confirmation model, four hypotheses were formulated in this study. The relationship between EC and PU was found to be non-significant (β = −0.025, *p* = 0.374), suggesting that EC does not significantly influence users' perceptions of the usefulness of the service. In contrast, EC had a significant positive effect on SS (β = 0.356, *p* < 0.001), indicating that users' satisfaction with the service is strongly influenced by how well their expectations are met. Further, the relationship between PU and SS was also significant (β = 0.219, *p* < 0.001), further reinforcing the idea that the perceived value and utility of the service directly contribute to users' satisfaction. This finding underscores the essential role of PU in shaping users' satisfaction levels. The relationship between SS and CI was particularly strong, with a path coefficient of 0.791 (*p* < 0.001). This suggests that users who are satisfied with the service are highly likely to continue using it, highlighting the critical role of SS in determining users' future engagement with the platform. Therefore, H2, H3, and H4 are supported.

For the expanded framework Information System Success Model, three hypotheses were formulated in this study. Both IQ, SysQ and SQ were found to significantly affect PU. IQ, had a path coefficient of 0.234 (*p* < 0.001), SysQ had a path coefficient of 0.365 (*p* < 0.001), while SQ had a coefficient of 0.286 (*p* < 0.001). These findings emphasize the crucial role of both information, system and SQ in enhancing users' perceptions of the service's usefulness, underscoring the importance of providing a reliable and high-quality experience. Therefore, H5, H6, and H7 are supported, affirming the validity of applying the Information System Success Model in this study. These results confirm the effectiveness of utilizing the Information System Success Model to extend the understanding of PU in this study. These findings provide strong empirical evidence that the model is a valid framework for exploring the factors that influence users' perceptions of usefulness, thereby enhancing the robustness of the theoretical foundation in this research.

Additionally, IQ was found to significantly influence EC (β = 0.234, *p* < 0.001), highlighting the importance of high-quality information in shaping users' expectations.

Finally, PFP emerged as a strong determinant of both EC (β = 0.587, *p* < 0.001) and SS (β = 0.427, *p* < 0.001), suggesting that users' perceptions of fair pricing are critical to confirming their expectations and enhancing their satisfaction with the service. This finding is consistent with the broader understanding that perceived fairness in pricing plays a crucial role in shaping users' overall experiences.

#### 4.2.4 Indirect effects

Indirect effects include total indirect effects and specific indirect effects. Total indirect effects are the effects of one variable on another variable through one or more mediating variables. It considers all possible indirect paths between variables, reflects the interactions between variables through mediating variables, reveals the complex relationships between variables through mediating variables, and helps us to understand how variables interact with each other through potential mediating mechanisms. The model comprises a total of 13 total indirect effects. Of these, 11 total indirect effects were found to be statistically significant (*p* < 0.01). Specifically, the total indirect effects between EC and CI (β = 0.277, *p* < 0.001), IQ and CI (β = 0.119, *p* < 0.001), PFP and CI (β = 0.500, *p* < 0.001), PU and CI (β = 0.173, *p* < 0.001), SQ and CI (β = 0.049, *p* < 0.001), and SysQ and CI (β = 0.063, *p* < 0.001) all showed significant positive effects. These findings suggest that EC, IQ, PFP, PU, SQ, and SysQ have substantial and significant total indirect effects on CI in music streaming services.

Moreover, the total indirect effects between IQ and SS (β = 0.150, *p* < 0.001), PFP and SS (β = 0.205, *p* < 0.001), and SQ and SS (β = 0.063, *p* < 0.001) were also statistically significant, indicating that these factors positively influence SS.

However, the total indirect effects between EC and SS (β = −0.005, *p* = 0.379), IQ and PU (β = −0.006, *p* = 0.392), and PFP and PU (β = −0.015, *p* = 0.369) did not reach statistical significance (*p* > 0.05). These results suggest that while these variables influence other aspects of the model, their total indirect effects on SS and PU are not significant. For instance, EC has a significant positive path coefficient (β = 0.356, *p* < 0.001) to SS, and IQ demonstrates a significant positive path coefficient (β = 0.314, *p* < 0.001) to PU. However, despite these significant direct effects, the total indirect effects involving these variables through other mediating factors do not provide statistically significant contributions to SS and PU.

Specific indirect effects are the effects of one variable on another through specific mediating variables or pathways. Specific indirect effects describe the contribution of each specific indirect pathway to the total indirect effect across all possible indirect relationships. The model includes 21 specific indirect effects, of which 13 are statistically significant (*p* < 0.01). The results of specific indirect effects (shown in Table 6) highlight that several specific indirect effects, particularly those involving IQ, PFP, and SysQ, play a crucial role in driving user satisfaction and CI. However, the analysis also reveals that eight specific indirect effects are not statistically significant (*p* > 0.05). A closer inspection reveals that all non-significant specific indirect effects share the common pathway EC → PU. This suggests that once users' expectations are confirmed, the pathway through PU does not have a significant impact on SS or CI in this context. The EC → PU path itself has a non-significant coefficient (β = −0.025, *p* = 0.374), further supporting the idea that confirmation of expectations does not substantially enhance PU. This may imply that while users' expectations play an important role in their overall satisfaction, the PU of the service is not significantly influenced by EC in this particular study.

**Table 6:** Specific indirect effects.

**Path**	**Original sample**	**Sample mean**	**Standard deviation**	**T statistics**	***P* values**
PFP -> EC -> PU -> SS -> CI	−0.003	−0.002	0.003	0.886	0.376
EC -> PU -> SS	−0.005	−0.005	0.006	0.879	0.379
IQ -> PU -> SS	0.069	0.069	0.013	5.236	0.000
IQ -> EC -> PU	−0.006	−0.006	0.007	0.857	0.392
PFP -> EC -> PU	−0.015	−0.014	0.016	0.899	0.369
IQ -> EC -> SS	0.083	0.084	0.016	5.053	0.000
SQ -> PU -> SS	0.063	0.063	0.012	5.177	0.000
PFP -> EC -> SS	0.209	0.210	0.028	7.535	0.000
SysQ -> PU -> SS	0.080	0.080	0.011	7.027	0.000
SysQ -> PU -> SS -> CI	0.063	0.063	0.009	6.774	0.000
SQ -> PU -> SS -> CI	0.049	0.050	0.010	5.052	0.000
IQ -> EC -> PU -> SS	−0.001	−0.001	0.001	0.847	0.397
PFP -> EC -> PU -> SS	−0.003	−0.003	0.004	0.888	0.374
IQ -> EC -> PU -> SS -> CI	−0.001	−0.001	0.001	0.844	0.399
EC -> PU -> SS -> CI	−0.004	−0.004	0.005	0.877	0.381
IQ -> PU -> SS -> CI	0.054	0.055	0.011	5.147	0.000
IQ -> EC -> SS -> CI	0.066	0.066	0.013	5.033	0.000
PFP -> EC -> SS -> CI	0.165	0.166	0.022	7.646	0.000
EC -> SS -> CI	0.281	0.283	0.034	8.222	0.000
PFP -> SS -> CI	0.338	0.338	0.032	10.529	0.000
PU -> SS -> CI	0.173	0.173	0.021	8.108	0.000

#### 4.2.5 Total effects

Total effects reflect the full impact of one variable on another and are a comprehensive assessment of all possible effects (direct and indirect) of a variable (Mohammadi et al., 2023). Based on the results in Table 7, the model includes 20 total effects, of which 19 are statistically significant (*p* < 0.01). Only two total effects are not statistically significant (*p* > 0.05). The results highlight the strong influence of IQ, PFP, SQ, and SysQ on SS and CI, with most total effects showing significant impacts. PU, as anticipated, serves as a key mediator for several relationships, affecting both satisfaction and retention. However, the non-significant effects related to EC → PU (β = −0.025, *p* = 0.374) and PFP → PU (β = −0.015, *p* > 0.05) suggest that while these factors influence other aspects of user behavior, their direct impact on PU may be limited. This may warrant further investigation to explore how different contexts or models might affect these relationships.

**Table 7:** Total effects.

**Path**	**Original sample**	**Sample mean**	**Standard deviation**	**T statistics**	***P* values**
EC -> CI	0.277	0.279	0.035	7.926	0.000
EC -> PU	−0.025	−0.024	0.028	0.890	0.374
EC -> SS	0.350	0.352	0.045	7.795	0.000
IQ -> CI	0.119	0.120	0.014	8.343	0.000
IQ -> EC	0.234	0.234	0.033	7.092	0.000
IQ -> PU	0.308	0.308	0.041	7.519	0.000
IQ -> SS	0.150	0.151	0.018	8.544	0.000
PFP -> CI	0.500	0.502	0.023	21.348	0.000
PFP -> EC	0.587	0.588	0.031	18.767	0.000
PFP -> PU	−0.015	−0.014	0.016	0.899	0.369
PFP -> SS	0.632	0.633	0.023	27.419	0.000
PU -> CI	0.173	0.173	0.021	8.108	0.000
PU -> SS	0.219	0.218	0.026	8.515	0.000
SQ -> CI	0.049	0.050	0.010	5.052	0.000
SQ -> PU	0.286	0.287	0.044	6.523	0.000
SQ -> SS	0.063	0.063	0.012	5.177	0.000
SS -> CI	0.791	0.793	0.019	41.065	0.000
SysQ -> CI	0.063	0.063	0.009	6.774	0.000
SysQ -> PU	0.365	0.365	0.035	10.560	0.000
SysQ -> SS	0.080	0.080	0.011	7.027	0.000

## 5 Discussion

This study extends the expectation confirmation model by integrating four additional variables to examine satisfaction and CI within the context of music streaming services. The analysis provides several important insights into the key factors that drive user retention. Specifically, the results underscore the central role of SS in influencing CI, the significant impact of PU and EC, and the importance of other factors such as IQ, PFP, SysQ, and SQ. Understanding these relationships allows service providers to better tailor their offerings to enhance user experiences and foster greater loyalty.

Regarding SS, the study reaffirms that it remains the core determinant of CI. With a strong path coefficient, SS directly influences users' intention to continue using the service, reinforcing the notion that positive user experiences are critical for sustaining long-term engagement. This finding is consistent with previous research that has highlighted the centrality of satisfaction in promoting consumer loyalty and retention (Aityassine, 2022; Erjavec et al., 2016; Omoregie et al., 2019). When users are satisfied with the service, they are far more likely to remain loyal, suggesting that maintaining a high level of SQ is crucial for retention (Mittal, 2016).

PU also plays a pivotal role in shaping SS, which, in turn, influences CI (Chen and Li, 2017). The significant positive relationship between these variables suggests that users who find the service useful are more likely to be satisfied, which ultimately encourages them to continue using the service. This is particularly relevant in the context of music streaming services, where PU is integral to maintaining user engagement (Lee et al., 2021). A service that is perceived as highly useful not only meets users' expectations but also enhances their overall experience, making it more likely they will continue using the service (Nan et al., 2020).

Similarly, EC influences SS and, subsequently, CI (Alsadoon, 2022). This finding is consistent with the research of Arteaga-Sánchez et al. (2020), which emphasizes the importance of aligning service delivery with user expectations to foster satisfaction, which directly strengthens the likelihood of continued use. However, it is noteworthy that EC did not have a significant effect on PU in this study. This deviation from the traditional expectation confirmation model framework, where EC is expected to positively influence PU (Brown et al., 2008), can be attributed to the unique characteristics of music streaming services. In these services, user expectations are more often driven by content availability and the overall user experience rather than the platform's functional utility (Barata and Coelho, 2021). Similar results have been observed in studies of other digital services, where the link between EC and PU is less pronounced (Nematolahi et al., 2017). Moreover, in the current era of intense competition in the music streaming market, where numerous services offer similar features and functionalities, users have developed comparable expectations after prolonged use (Morris and Powers, 2015). As music streaming platforms increasingly offer similar functionalities, users may focus less on functionality and more on content variety, user experience, and additional non-functional features (Barata and Coelho, 2021; Ferwerda et al., 2019). This shift in focus could explain why EC has a reduced impact on PU, as users come to expect a baseline level of functionality across services. Consequently, the role of EC in shaping overall user satisfaction and retention becomes more prominent, while its effect on PU diminishes in a highly competitive and functionally converging market.

In addition, the study highlights the significant role of PFP. PFP showed a strong direct effect on SS, which subsequently influenced CI (Cakici et al., 2019; Moriuchi and Murdy, 2024). This finding is consistent with the research of Ng et al. (2022), which suggests that when users perceive the pricing of a service as fair, it not only affects their perception of value but also significantly contributes to their satisfaction and long-term retention. Furthermore, PFP is identified as a major driver of EC (Banerjee et al., 2024). The robust relationship between these two variables indicates that fair pricing plays a crucial role in confirming user expectations about the service, which is essential for fostering a positive user experience.

The findings also suggest that IQ is a critical factor influencing PU. The significant and positive path coefficient between IQ and PU implies that high-quality, relevant, and up-to-date information enhances the perceived value of the music streaming service. This finding aligns with the research of Priyadarshini et al. (2017), which also highlights the importance of IQ in enhancing user perceptions of value and usefulness in digital services. This, in turn, encourages continued use, highlighting the importance of providing accurate and comprehensive content to maintain user engagement. Users who feel that the information provided by the service is valuable are more likely to perceive the service as useful, which strengthens their overall satisfaction (Shim and Jo, 2020).

Additionally, the results indicate that SysQ positively influences both PU and SS. A well-functioning platform that offers a seamless user experience enhances the PU of the service, which leads to greater user satisfaction (Chiang et al., 2019; Salim et al., 2021). This, in turn, positively effects CI, underlining the importance of robust system performance and ease of use in fostering user retention (Al-Hattami and Almaqtari, 2023; Masri et al., 2020).

Lastly, SQ plays a significant role in shaping PU. As SQ improves, users are more likely to perceive the service as valuable, which in turn enhances both their satisfaction and their intention to continue using the service (Chikazhe and Nyakunuwa, 2022; Sharma et al., 2024). This result highlights the critical importance of high-quality service delivery across all aspects. A service that consistently meets or exceeds user expectations in terms of quality is more likely to retain its users over time (Xin et al., 2023).

## 6 Implications

### 6.1 Theoretical implications

Theoretically, this study makes several important contributions to the literature on user retention in digital services, specifically in the music streaming industry. By extending the expectation confirmation model framework, the research provides a deeper understanding of how PU, EC, SS, and other key factors interact to influence CI. The inclusion of additional variables such as IQ, PFP, SysQ, and SQ enriches the Expectation confirmation model and offers a more comprehensive theoretical model that accounts for the complexities of user behavior in digital service environments.

Furthermore, this study challenges some of the traditional assumptions of the Expectation confirmation model, particularly the relationship between EC and PU. The finding that EC does not exert a significant influence on PU in the music-streaming context. This outcome differs from the positive EC to PU association commonly reported in earlier ECM work across e-commerce and e-learning (Al Amin, 2022; Li et al., 2024). One possible reason is that music-streaming platforms are highly hedonic and content-centric, leading users to place greater value on entertainment, habitual engagement, and catalog breadth than on functional utility. Supporting this interpretation, Helkkula (2016) study of paid “Music-as-a-Service” subscriptions showed that hedonic motivation, price value, and habit explained a large share of behavioral intention, whereas PU was not a significant predictor. Taken together, the evidence indicates that the traditional EC to PU pathway may be attenuated when experiential gratifications overshadow utilitarian considerations in digital music services. This deviation opens up new avenues for rethinking the role of EC in digital service contexts where functionalities across competing services are increasingly similar.

### 6.2 Practical implications

From a practical perspective, the insights gained from this study provide valuable guidance for music streaming service providers aiming to enhance user satisfaction and increase retention. The finding that SS is the most significant determinant of CI underscores the importance of delivering a satisfying user experience. Service providers should focus on improving elements that directly contribute to user satisfaction, such as content variety, platform performance, and ease of use.

Moreover, the study highlights the importance of PFP in shaping EC and SS. This suggests that pricing strategies should not only reflect the perceived implications of the service but also align with users' expectations. Transparent and fair pricing models can lead to higher satisfaction, which directly influences retention.

The role of IQ, SysQ, and SQ further suggests that service providers must prioritize high-quality content, seamless system performance, and overall service delivery. Given the competitive nature of the music streaming market, where many platforms offer similar functionalities, differentiating based on content quality, system reliability, and high-quality service can serve as key levers for sustaining long-term user engagement.

### 6.3 Limitations and future research

While this study provides valuable insights, several limitations must be acknowledged. First, the research is cross-sectional in nature, which limits the ability to draw causal inferences over time. Future studies could adopt a longitudinal approach to better understand the long-term dynamics of user satisfaction and CI in music streaming services. Longitudinal data would allow researchers to observe how changes in service offerings, user experience, or market conditions affect user behavior over an extended period.

Second, this study specifically focuses on the Chinese market, which limits the generalizability of the findings to other cultural contexts. The unique characteristics of Chinese users, such as cultural preferences, consumption patterns, and attitudes toward digital services, may influence their expectations and satisfaction with music streaming services. As a result, the study's findings may not fully apply to users in other regions or cultures. Future research could replicate this study in different cultural or regional contexts to explore whether the identified factors influencing user retention differ across markets. Comparative studies could also investigate how user preferences and expectations vary in regions with distinct music consumption habits.

## Data Availability

The raw data supporting the conclusions of this article will be made available by the authors, without undue reservation.
